# The prevalence and clinical correlates of medical disorders comorbidities in patients with bipolar disorder

**DOI:** 10.1186/s12888-022-03819-0

**Published:** 2022-03-10

**Authors:** Zhonggang Wang, Tao Li, Shuhua Li, Kunkun Li, Xianfei Jiang, Chen Wei, Lei Yang, Haiyan Cao, Shen Li, Jie Li

**Affiliations:** 1 Department of Psychiatry, Shandong Daizhuang Hospital, Jining, 272051 Shandong China; 2 Institute of Mental Health, Tianjin Anding Hospital, Mental Health Center of Tianjin Medical University, Tianjin, 300222 China; 3 Department of Clinical Psychology, Tianjin TEDA Hospital, Tianjin, 300457 China; 4 Department of Psychiatry, Affiliated Xuzhou Oriental Hospital of Xuzhou Medical University, Xuzhou, 221004 Jiangsu China; 5 Institute of Mental Health, Tianjin Disizhongxin Hospital, Tianjin Medical University, 3 Zhongshan Rd., Hebei District, Tianjin, 300142 China; 6 Department of Psychiatry, School of Mental Health, Jining Medical University, Jining, 272191 Shandong China; 7 Department of Psychiatry, College of Basic Medical Sciences, Tianjin Medical University, Tianjin, 300070 China

**Keywords:** Bipolar disorder, Medical disorders, Comorbidity prevalence, Clinical features and correlates

## Abstract

**Objective:**

Medical disorders in patients with bipolar disorder (BD) have attracted more and more attention. So far, there is still a lack of studies on this issue utilizing large sample sizes in the Chinese sample. Therefore, we conducted this study to explore the clinical characteristics of BD patients comorbid medical disorders in a relatively large Chinese sample.

**Methods:**

This was a cross-sectional study including 1,393 BD patients (882 patients with medical disorders and 511 patients without medical disorders). Their demographic and clinical characteristics were obtained by the Hospital Information System and self-designed questionnaires.

**Results:**

The comorbidity rate of medical disorders in BD was 63.32%. The average number of medical disorders for a BD patient was 2.69. The top five comorbid medical disorders in BD patients were circulatory system diseases (19%), nervous system diseases (18%), endocrine and metabolic diseases (17%), digestive system diseases (16%), and respiratory system diseases (8%). BD patients with comorbid medical disorders had an older average age, lower education level, longer illness course, later onset age, lower ratio of psychotic features, more admission numbers, higher ratio of smoking and drinking alcohol, more number of manic episodes (All *P* < 0.05). Smoking, numbers of depressive episode, onset age, and illness course were independent risk factors of comorbidities in BD patients (All *P* < 0.05).

**Conclusions:**

Medical disorders in Chinese BD patients are highly prevalent. The smoking, number of depressive episodes, onset age, illness course, are correlated with BD patients comorbid medical disorders. Clinicians should pay attention to the medical disorders comorbidities in BD patients, and take effective measures to improve treatment outcome and reduce the suffering. The integrative approach should be the imperative in clinical practice.

## Introduction

Bipolar disorder (BD) is a common, complex, and recurrent severe mental disorder. The quality of life of BD patients and their families is seriously affected. Nowadays, comorbidities of BD patients have become a topic of increasing concern for psychiatrists and researchers. Comorbidities increases the difficulties of treatment and affects the prognosis of BD. The central principle of clinical comorbidity is that there are two or more kinds of syndromes in the same patient, which belong to different classified entities [1]. The comorbidities of BD could worsen the illness course, delay rehabilitation, prolong hospitalization, increase recurrence and the risk of suicide, and reduce the quality of life of patients [2]. One study showed that comorbidity with physical conditions could worsen social and cognitive functions of BD patients [3]. Comorbidities can have an important effect on the choice of drugs for the treatment of BD patients, as the possible drugs interactions with ongoing medications for medical disorders [4]. Moreover, it has been reported that the average age of death in patients with severe mental disorders such as BD is 10 years earlier than that of the general population, and the high rate of comorbidity was a significant risk factor [5].

A previous study showed that the comorbidity rate of BD and various medical disorders was about 96.3% [6]. Another study showed that more than 60% of BD patients have a comorbid diagnosis of various medical disorders [4]. Additionally, Jolfaei et al. found that the prevalence of BD in a population of patients with medical disorders was far more frequent than that of the general population [7]. The medical disorders linked with BD include many systemic diseases, and a BD patient may suffer from multiple medical disorders. At present, it has been reported that the comorbidities of BD include brain diseases, cardiovascular diseases, respiratory diseases, genitourinary diseases, endocrine diseases, blood system diseases, infectious diseases, and other system diseases in studies and clinical practice [8, 9]. The association between BD and medical disorders comorbidities further seems to be bidirectional [10].

Many studies have investigated the characteristics of BD patients comorbid medical disorders [11–13]. A study found that young BD patients experienced higher comorbid rates of several diseases, which were linked to risk factors such as engagine in unhealthy lifestyle behaviors, experiencing worse medication side effects, receiving poorer health care services, biologic susceptibility, and socio-economic status [14]. Furthermore, age is another impact factor, and a study found that medical disorders comorbidities in BD patients increased with age [8]. Some researchers supposed that basic biological factors, genetic factors, and environmental stresses were probable explanations for the high comorbidity rate of BD and physical illnesses [7, 15, 16]. Studies on the neurobiology of BD with comorbid medical diseases showed that changes in serotonin and dopamine were important influencing factors as well [7, 17]. An outbreak and increase of dopamine were also found in some medical disorders, indicating that medical disorders and BD may overlap with a common neurobiological etiology [18].

To the best of our knowledge, there is still less study exploring the prevalence and associated factors of comorbid medical disorders in patients with BD in a Chinese sample. Therefore, the aims of our study were: 1) to explore the prevalence rate of medical disorders of patients with BD in 1,393 Chinese participants; 2) to find the associated factors of comorbid medical disorders in BD patients.

## Participants and methods

### Study participants

There were 1,393 BD inpatients recruited from Shandong Daizhuang Hospital in China between January 1, 2018 and December 31, 2018. Inclusion criteria were as follows: 1) Primary diagnosis meeting the BD diagnosis based on International Classification of Diseases-10(ICD-10) criteria, confirmed by two experienced psychiatrists; 2) no age limits; 3) having at least middle school education; 4)Han ethnicity, Chinese; 5) negative pregnancy test for women. Exclusion criteria were as follows: 1) a lifetime diagnosis of intravenous drug dependency; 2) experiencing affective illness secondary to physical illness or medication.

In this study, each BD patients were given the related laboratory tests and examinations after enrollment. Medical disorders were also diagnosed by physician according to ICD-10 criteria. All medical disorders were sorted into twelve system diseases, including circulatory system diseases, nervous system diseases, endocrine, nutritional, and metabolic diseases, digestive system diseases, respiratory system diseases, genitourinary disease, neoplasms diseases, diseases of the blood and blood - forming organs, injury and poisoning diseases, infectious diseases, diseases of musculoskeletal system, connective tissue diseases, and diseases of the skin and subcutaneous tissue. Some diseases were sorted into other system diseases.

All patients were divided into two groups according to whether they had medical disorders comorbidities or not. 882 BD patients with at least one medical disorders were in the comorbidity group. There were 458 males and 424 females with an average age of (42.09±15.40) years and an average education level of (8.74±4.04) years. Patients without any types of medical disorders were in the non-comorbidity group (*n*=511). This group included 241 males and 270 females with an average age of (35.42±13.95) years and an average education level of (9.46±3.87) years. Demographic characteristics are described in Table 1.

**Table 1:** Demographic and clinical characteristics of all participants studied

Variables	Values
Age(years, mean ± SD)	39.64 ± 15.22
Sex [n (%)]	
Males	699(50.18%)
Females	694(49.82%)
Education level(years, mean ± SD)	9.00 ± 3.99
Nation [n (%)]	
Han	1381(99.14%)
Other	12(0.86%)
Comorbidity [n (%)]	882(63.32%)
Non-comorbidity [n (%)]	511(36.68%)
Average number of physical comorbidities per patient	2.69 ± 1.83

Values are expressed as the n (%) or mean ± standard deviation

This study was approved by Shandong Daizhuang Hospital ethics committee. All methods were performed in accordance with the relevant guidelines and regulations of the ethics committee. 

### Demographics and clinical variables collection

The data for this study were collected through self-designed questionnaires and based on the Hospital Information System. The demographic variables collected were as follows: age (years), sex, race, education (years), occupation status, address, and marital status.

In addition, the clinical variables included: illness course, onset age, onset symptom type of first affective disorder, family history of mental disorders, psychotic features, suspected precipitating event for index event, premorbid personality, smoking history, alcohol history, number of inpatient admissions, length of stay, revisiting times, suicide attempt, suicide behavior, age of onset manic episode, age of onset depressive episode, numbers of manic episodes, numbers of depressive episodes, names and types of comorbid medical disorders, and number of comorbid medical disorders.

### Statistical analyses

All statistical analyses were performed using IBM Statistical Package for the Social Sciences (SPSS) Version 20.0 software. Demographic and clinical characteristics as well as types and number of comorbid medical disorders were compared between BD patients in the comorbidity group and non-comorbidity group. Age, education level, illness course, age of onset, revisiting times, length of stay, and admission numbers were described by (mean ± SD) as continuous variables. Independent sample t-tests were used to compare age, education level, illness course, age of onset, revisiting times, and length of stay between the two groups. Rank-sum test was used to compare admission numbers. Marriage, occupation, family history of mental disease, sex, onset form, suicide attempt and behavior, and psychotic features were described by (n, %) as categorical variables. Chi-Square tests were used to analyze the above variables between the two groups. Logistic regression analysis was used to test the independent associated factors affecting comorbidities of patients with BD. Comorbidities/non-comorbidities was a dependent variable. Clinical characteristics data were independent variables, including illness course, onset age, psychotic features, admission numbers, smoking, drinking alcohol, onset age of manic episode, age of onset depressive episode, numbers of manic episodes, and numbers of depressive episodes. For all analyses, *p* value < 0.05 for two tails was used as a reference to determine the statistical significance test result.

## Results

### Characteristics of participants

The demographic and clinical characteristics of the participants were summarized in Table 1. In 1,393 BD patients, 882 (63.32%) patients had comorbid medical disorders; 511 (36.68%) patients did not have comorbid medical disorders. The average number of comorbid medical disorders per patient with BD was 2.69.

### Comparisons for demographic characteristics

Comparisons of demographic characteristics between the two groups were summarized in Table 2. BD patients with comorbid medical disorders had an older average age (*t* = 8.28; *p* < 0.01), lower education level (*t* = -3.27; *p* < 0.01), and worse marriage state (*χ*^*2*^ = 19.76; *p* < 0.01).There were no significant differences between the two groups regarding occupation, race, address, or sex (all *p* > 0.05).

**Table 2:** Comparison of demographic characteristics between the two groups

	BD with comorbid medical disorders(*n* = 882,63.32%)	BD without comorbid medical disorders(*n* = 511,36.68%)	χ^2^/t -value	*P*-value
Age				
(years, mean ± SD)	42.09 ± 15.40	35.42 ± 13.95	8.28	< 0.01^**^
Education level				
(years, mean ± SD)	8.74 ± 4.04	9.46 ± 3.87	-3.27	< 0.01^**^
Marriage [n (%)]			19.76	< 0.01^**^
Unmarried	197(22.34%)	169(33.07%)		
Married	595(67.57%)	303(59.30%)		
Divorce/separated/widowed	89(10.09%)	39(7.63%)		
Occupation [n (%)]			6.74	= 0.08
Worker	145(16.44%)	84(16.44%)		
Farmer	305(34.58%)	144(28.18%)		
Student	412(46.72%)	269(52.64%)		
Other	20(2.27%)	14(2.74%)		
Race [n (%)]			^a^	= 0.23
Han	872(98.87%)	509(99.61%)		
Other	10(1.13%)	2(0.39%)		
Address [n (%)]			0.56	= 0.45
Urban,	278(31.52%)	171(33.46%)		
Rural	604(68.48%)	340(66.54%)		
Sex [n (%)]			2.94	= 0.09
Male	458(51.93%)	241(47.16%)		
Female	424(48.07%)	270(52.84%)		

^a^is Fisher’s exact test; **p < 0.01

### Comparisons for clinical characteristics

Clinical characteristics were summarized in table 3. There were significant differences in illness course (*t* =-5.77; *p* < 0.01), onset age ( *t* = 5.57; *p* < 0.01), ratio of psychotic features (*χ*^*2*^ = 5.62; *p* = 0.02), admission numbers (*t* = 2.12; *p* = 0.03), ratio of smoking (χ^2^ = 9.60; *p* < 0.01), ratio of drinking alcohol (*χ*^*2*^ = 4.68; *p* = 0.03), age of onset manic episode (*t* = (t=5.51); *p* < 0.01), age of onset depressive episode (*t* = 5.87; *p* < 0.01 ), number of manic episodes(*t* = 3.57; *p* < 0.01),or number of depressive episodes(*t* = 3.44; *p* < 0.01) between comorbidity patients and non-comorbidity patients. There were no significant differences in other variables between the two groups (all *p* >0.05).

**Table 3:** Comparison of clinical characteristics between the two groups

	BD with comorbid medical disorders(*n* = 882,63.32%)	BD without comorbid medical disorders(*n* = 511,36.68%)	χ^2^/t/z-value	*P*-value
Illness course	13.98 ± 11.41	10.63 ± 10.01	-5.52--【-5.77】	< 0.01**
Onset Age	28.32 ± 12.47	24.81 ± 10.62	5.34--【5.57】	< 0.01^**^
Onset Symptom			0.44	= 0.51
Mania/Hypomania	342(38.78%)	189(36.99%)		
Depression	540(61.22%)	322(63.01%)		
Family History of mental disorder			0.20	= 0.66
Positive	234(26.53%)	130(25.44%)		
Negative	648(73.47%)	381(74.56)		
Onset Form			0.22	= 0.90
acute	280(31.75%)	160(31.31%)		
Sub-acute	103(11.68%)	64(12.52%)		
chronic	499(56.58%)	287(56.16%)		
Psychotic features			5.62	= 0.02^*^
Yes	372(42.18%)	249(48.73%)		
No	510(57.82%)	262(51.27%)		
Admission numbers	3.21 ± 2.68	2.87 ± 2.43	2.12	= 0.03
Length of stay Last admission	48.49 ± 51.10	45.58 ± 35.69	1.13	= 0.26
Suspected precipitating event for index event			1.49	= 0.22
Yes	199(22.56%)	130(25.44%)		
No	683(77.44%)	381(74.56%)		
Premorbid personality			2.38	= 0.30
Introverted	617(69.95%)	371(72.60%)		
Extroverted	246(27.89%)	134(26.22%)		
Neutral	19(2.15%)	6(1.17%)		
Smoking			9.60	< 0.01^**^
Yes	173(19.61%)	67(13.11%)		
No	709(80.39%)	444(86.89%)		
Drinking alcohol			4.68	= 0.03^*^
Yes	99(11.22%)	39(7.63%)		
No	783(88.78%)	472(92.37%)		
Suicide attempt			0.04	= 0.85
Yes	336(38.10%)	192(37.57%)		
No	546(61.90%)	319(62.43%)		
Suicide behavior			0.08	= 0.78
Yes	140(15.87%)	84(16.44%)		
No	742(84.13%)	427(83.56%)		
Revisiting Times	19.98 ± 20.35	20.93 ± 20.56	-0.84	= 0.40
Age of onset manic episode	30.91 ± 14.13	27.07 ± 11.51		< 0.01^**^
Age of onset depressive episode	29.91 ± 13.36	25.63 ± 11.27	5.87	< 0.01^**^
Numbers of manic episode	2.79 ± 2.76	2.30 ± 1.94		< 0.01^**^
Numbers of depressive episode	2.29 ± 2.39	1.91 ± 1.62		< 0.01^**^

^*^p < 0.05, **p < 0.01

### Types and distribution of comorbid medical disorders in patients with BD

**Fig. 1:**
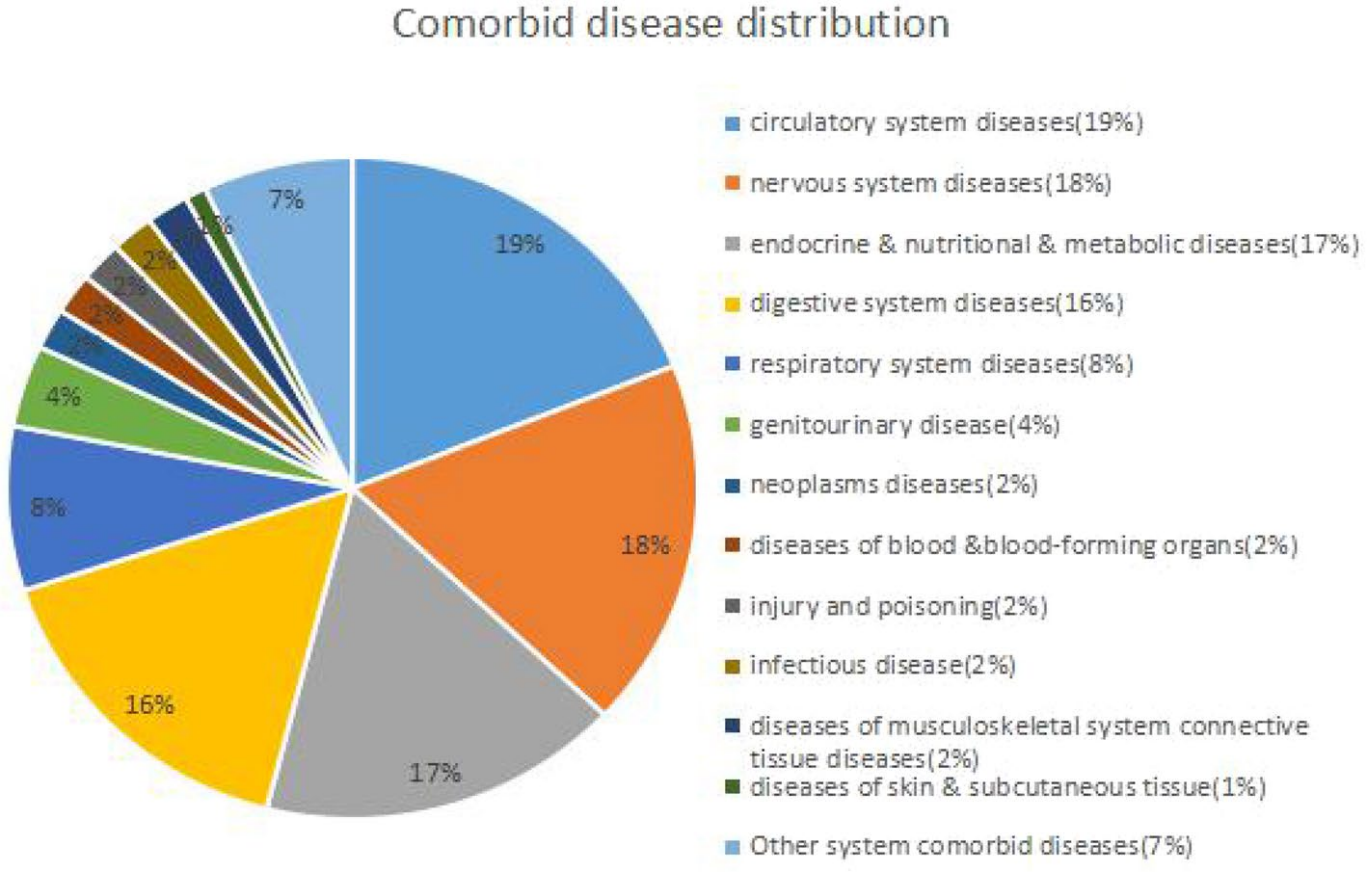
Comorbid medical disorders distribution in BD patients

Types and distribution of comorbid medical disorders were summarized in Fig. 1. In order of decreasing prevalence, the various types of comorbid medical disorders present in the comorbidity group were circulatory system diseases (19%), nervous system diseases (18%), endocrine, nutritional, and metabolic diseases (17%), digestive system diseases (16%), respiratory system diseases (8%), genitourinary disease (4%), neoplasms diseases (2%), diseases of the blood and blood-forming organs (2%),injury and poisoning (2%), infectious disease (2%), diseases of musculoskeletal system connective tissue diseases (2%), and diseases of the skin and subcutaneous tissue (1%). Other system comorbid diseases with BD were 7% of the total cormorbid diseases.

**Fig. 2:**
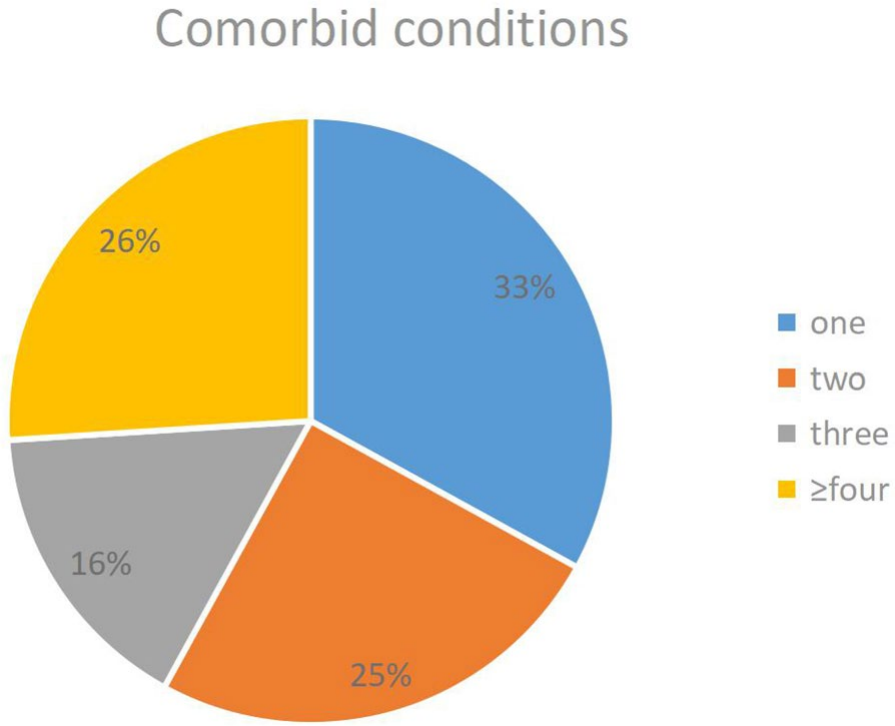
Comorbid medical disorders number of per BD patient

Figure 2 shows that the distribution of patients who suffered from medical disorders from one or multiple systems. 33% patients with BD suffered from one system of medical disorders. 26% patients suffered from more than four systems of medical disorders. 25% patients suffered from two systems of medical disorders. 16% patients suffered from three systems of medical disorders.

### Associated factors with medical disorders comorbidities in BD patients

Results from the logistics regression analysis of clinical factors affecting comorbidities in patients with BD are found in Table 4. Table 4 shows that illness course, onset age, smoking, and number of depressive episodes were significant risk factors of comorbidities in BD patients (all *p* < 0.05). Among the above risk factors, illness course was the most strongly associated factor (*OR* = 1.031, *p* < 0.001). According to the effects on comorbidities, other risks were smoking (*OR* = 1.663, *p* = 0.012), number of depressive episodes (*OR* = 1.092, *p* = 0.031), and onset age (*OR* = 1.044, *p* = 0.048).

**Table 4:** Logistics regression analysis of clinical factors affecting comorbidities in patients with bipolar disorder

	B	SE	*P*-value	*OR*	95% *CI* for *OR*	
					Lower	Upper
Illness course	0.031	0.008	< 0.001	1.031	1.016	1.047
Onset Age	0.043	0.022	0.048	1.044	1.000	1.090
Psychotic features	-0.186	0.127	0.143	0.830	0.647	1.065
Admission numbers	-0.033	0.033	0.324	0.968	0.907	1.033
Smoking	0.508	0.203	0.012	1.663	1.116	2.477
Drinking alcohol	-0.113	0.266	0.671	0.893	0.531	1.503
Age of onset manic episode	-0.009	0.013	0.472	0.991	0.966	1.016
Age of onset depressive episode	-0.004	0.015	0.781	0.996	0.968	1.025
Numbers of manic episode	0.072	0.041	0.076	1.075	0.992	1.165
Numbers of depressive episode	0.088	0.041	0.031	1.092	1.008	1.182

Significant *p* values ≤ 0.05 at 95% confidence interval. Odds Ratio generated by binomial logistic regression model

*OR* Odds Ratio, *CI* Confidience interval

## Discussion

In this study, we analyzed the demographic and clinical characteristics of BD patients with comorbid medical disorders in a large sample of the Chinese sample. We found that: 1) there was a high prevalence of medical disorders comorbidities in Chinese BD patients; 2)the average age of BD patients with comorbid medical disorders was higher than patients without comorbid medical disorders; 3) BD patients with comorbid medical disorders had a longer average illness course and later onset age than patients without comorbid medical disorders; 4) the most strongly associated factors affecting the comorbidity in BD patients were illness course, onset age, smoking, and number of depressive episodes.

Our results show that the comorbidity rate is higher among older BD patients, which was consistent with previous study [19]. Sylvia [20] found that 96.3% of BD patients had at least one medical disorders comorbidity. Older age BD patients had a greater likelihood of suffering from a cardio-metabolic condition. A possible reason for this finding is that physical functioning of BD patients with medical disorders decreases with aging. The risk of suffering from medical disorders,such as heart diseases, is gradually increasing with age [20], and the BD patients in the comorbid group were older on average. A study [21] categorized the physical diagnoses of 1,379 BD patients from 2001 to 2002 through outpatient psychiatric clinics and found that physical comorbidity rate increased with aging.

Our study showed that the independent factors related to comorbidities in BD patients included illness course, older onset age, smoking, and number of depressive episodes. Illness course was the most strongly associated factor (*p* < 0.001, *OR*=1.031). This suggests that the incidence of comorbidity increases with the prolongation of the course of disease in BD patients. With the prolongation of the course of disease, BD patients are more likely to suffer from various medical disorders. In this study, the smoking rate of BD patients in the comorbid group was higher than that in the non-comorbid group. Logistic regression analysis further showed that smoking history was a significantly correlated factor of suffering medical disorders in BD patients. This finding is consistent with the conclusions of other studies [22, 23]. López-Ortiz et al [24] found comorbidity of BD with smoking is 66-82.5%. Smoking results in poorer prognosis and greater clinical seriousness of BD. The most effective treatment approach is pharmacological treatment in combination with psychological interventions in order to increase efficacy and improve prognosis in patients with BD with smoking.

Compared with no-comorbid group patients, BD patients with comorbid medical disorders had more readmission, more manic episodes, and higher numbers of depressive episodes. The finding suggests that comorbid medical disorders may aggravate the condition of BD patients and increase the recurrence rate of diseases. This is particularly detrimental to the prognosis of patients. The longer illness course of the comorbid group demonstrated that comorbid medical disorders had a negative effect on BD patients, and led to poorer prognosis. This finding is consistent with another study [25]. Other researchers also found that comorbidity impacts lifestyle, treatment adherence, course, and the prognosis of BD patients [7].

Our study found that there were fewer psychotic symptoms in BD patients in the comorbid group than in the non-comorbid group. The related body of research regarding this topic is limited. Pinis et al. [26] found the incidence of psychotic symptoms in bipolar patients with other comorbid mental disorders was higher than in BD comorbid with an Axis I disorder. This finding provides information on the relationship between psychotic symptoms and comorbidities in BD patients. This relationship is particularly complicated and needs to be further explored in the future research.

In our study, the order of medical disorders comorbidities in BD patients in decreasing prevalence were circulatory system diseases (19%), nervous system diseases (18%), endocrine, nutritional, and metabolic diseases (17%), digestive system diseases(16%), respiratory system diseases (8%), genitourinary disease (4%), neoplasms diseases (2%), diseases of blood and blood-forming organs (2%), injury and poisoning (2%), infectious diseases (2%), diseases of musculoskeletal system connective tissue diseases (2%), and diseases of the skin and subcutaneous tissue (1%). The previous study [21] also found that the most common medical disorders in BD outpatients were endocrine and metabolic diseases (13.6%), circulatory diseases (13.0%), and nervous system diseases and sense organs (10.7%). Both studies showed that common complications included circulatory system diseases, nervous system diseases, endocrine and metabolic diseases, and digestive system diseases. Both studies showed higher comorbidity rates of various medical disorders among BD patients. Another study [27] on geriatric patients with BD also found a higher prevalence of medical disorders comorbidities. The authors found significantly higher incidences of chronic fatigue syndrome, migraine, asthma, chronic bronchitis, multiple chemical sensitivities, hypertension, and gastric ulcers in BD patients. Chronic medical disorders were associated with more severe conditions, increased household and work maladjustment, and higher utilization of physical services in BD patients [2, 28, 29].

Our study found that the average number of comorbid medical disorders per patient was 2.69. The previous study [9] found that the average number of comorbid medical disorders per patient was 2.5. Our study was consistent with the previous study. This point emphasizes the fact that BD patients are prone to two or more medical disorders. The underlying mechanisms of comorbidity remain unknown, requiring further clinical and basic study. Therefore, we should pay attention to this point in clinical work and carry out corresponding diagnosis, treatment, and intervention. Optimizing treatment of comorbid medical disorders could help to improve the illness conditons of BD patients [9]. Some studies suggest that treatment and prevention of general medical disorders comorbidities in BD should include priority prescriptions for physical disorders and lifestyle modifications [6, 10].

## Conclusion

This study demonstrated a significantly high incidence of medical disorders in hospitalized BD patients. Compared with BD patients without comorbidities, patients with comorbidities were older in age, had lower education levels, higher smoking/drinking rates, longer illness courses, more frequent manic/depressive episodes, and more frequent readmission. Our management of BD patients requires correct diagnosis and understanding of comorbid medical disorders to help determine the appropriate treatment needed. Patients with BD require appropriate management for comorbidities, such as cardiovascular disease and other medical disorders.

### Limitations

There were some limitations to the current study. First, we found that a higher incidence of the comorbidities in BD patients, but we did not discuss the effect of comorbidity on the prognosis of BD. Second, this study was a cross-sectional study, so it is limited in its analytical modelling of the data, and is difficult to draw causal conclusions. Future studies using a high-quality longitudinal design will help to further elucidate the inter-relationships between medical disorders comorbidities in BD patients.

## Data Availability

The raw data generated and analyzed in this study are not publicly available due to the appropriate protection of patients’ personal information, but are available from the corresponding author on a reasonable request.
