# Correction to: The prevalence and clinical correlates of medical disorders comorbidities in patients with bipolar disorder

**DOI:** 10.1186/s12888-022-03871-w

**Published:** 2022-04-01

**Authors:** Zhonggang Wang, Tao Li, Shuhua Li, Kunkun Li, Xianfei Jiang, Chen Wei, Lei Yang, Haiyan Cao, Shen Li, Jie Li

**Affiliations:** 1 Department of Psychiatry, Shandong Daizhuang Hospital, Jining, 272051 Shandong China; 2 Institute of Mental Health, Tianjin Anding Hospital, Mental Health Center of Tianjin Medical University, Tianjin, 300222 China; 3 Department of Clinical Psychology, Tianjin TEDA Hospital, Tianjin, 300457 China; 4 Department of Psychiatry, Affiliated Xuzhou Oriental Hospital of Xuzhou Medical University, Xuzhou, 221004 Jiangsu China; 5 Institute of Mental Health, Tianjin Disizhongxin Hospital, Tianjin Medical University, 3 Zhongshan Rd., Hebei District, Tianjin, 300142 China; 6 Department of Psychiatry, School of Mental Health, Jining Medical University, Jining, 272191 Shandong China; 7 Department of Psychiatry, College of Basic Medical Sciences, Tianjin Medical University, Tianjin, 300070 China

**Correction to: BMC Psychiatry 22, 176 (2022)**

**https://doi.org/10.1186/s12888-022-03819-0**

Following publication of the original article [1], the authors identified that an affiliation was missing. The affiliation has been added and assigned to the authors.

Institute of Mental Health, Tianjin Anding Hospital, Mental Health Center of Tianjin Medical University, Tianjin, China, 300222.

The original article [1] has been corrected.
